# Epidemic prediction of dengue fever based on vector compartment model and Markov chain Monte Carlo method

**DOI:** 10.1186/s12859-021-04059-x

**Published:** 2021-11-08

**Authors:** Chien-Hung Lee, Ko Chang, Yao-Mei Chen, Jinn-Tsong Tsai, Yenming J. Chen, Wen-Hsien Ho

**Affiliations:** 1 Department of Public Health, College of Health Sciences, Kaohsiung Medical University, Kaohsiung, Taiwan; 2 Research Center for Environmental Medicine, Kaohsiung Medical University, Kaohsiung, Taiwan; 3 Department of Medical Research, Kaohsiung Medical University Hospital, Kaohsiung Medical University, Kaohsiung, Taiwan; 4 Office of Institutional Research and Planning Section, Kaohsiung Medical University, Kaohsiung, Taiwan; 5 Department of Internal Medicine, Kaohsiung Municipal Hsiao-Kang Hospital, Kaohsiung Medical University, Kaohsiung, Taiwan; 6 School of Nursing, Kaohsiung Medical University, Kaohsiung, Taiwan; 7 Superintendent Office, Kaohsiung Medical University Hospital, Kaohsiung, Taiwan; 8 Department of Computer Science, National Pingtung University, Pingtung, Taiwan; 9 Department of Healthcare Administration and Medical Informatics, Kaohsiung Medical University, Kaohsiung, Taiwan; 10 Management School, National Kaohsiung University of Science and Technology, Kaohsiung, Taiwan

**Keywords:** Dengue transmission, Vector-susceptible-infectious-recovered with exogeneity (VSIRX), Epidemic prevention timing

## Abstract

**Background:**

Dengue epidemics is affected by vector-human interactive dynamics. Infectious disease prevention and control emphasize the timing intervention at the right diffusion phase. In such a way, control measures can be cost-effective, and epidemic incidents can be controlled before devastated consequence occurs. However, timing relations between a measurable signal and the onset of the pandemic are complex to be discovered, and the typical lag period regression is difficult to capture in these complex relations. This study investigates the dynamic diffusion pattern of the disease in terms of a probability distribution. We estimate the parameters of an epidemic compartment model with the cross-infection of patients and mosquitoes in various infection cycles. We comprehensively study the incorporated meteorological and mosquito factors that may affect the epidemic of dengue fever to predict dengue fever epidemics.

**Results:**

We develop a dual-parameter estimation algorithm for a composite model of the partial differential equations for vector-susceptible-infectious-recovered with exogeneity compartment model, Markov chain Montel Carlo method, and boundary element method to evaluate the epidemic periodicity under the effect of environmental factors of dengue fever, given the time series data of 2000–2016 from three cities with a population of 4.7 million. The established computer model of “energy accumulation-delayed diffusion-epidemics” is proven to be effective to predict the future trend of reported and unreported infected incidents. Our artificial intelligent algorithm can inform the authority to cease the larvae at the highest vector infection time. We find that the estimated dengue report rate is about 20%, which is close to the number of official announcements, and the percentage of infected vectors increases exponentially yearly. We suggest that the executive authorities should seriously consider the accumulated effect among infected populations. This established epidemic prediction model of dengue fever can be used to simulate and evaluate the best time to prevent and control dengue fever.

**Conclusions:**

Given our developed model, government epidemic prevention teams can apply this platform before they physically carry out the prevention work. The optimal suggestions from these models can be promptly accommodated when real-time data have been continuously corrected from clinics and related agents.

## Background

Weather and environmental factors affect dengue transmission patterns through the interactive dynamics between mosquito ecology and vector–human transmission. Due to dengue fever is prevalent in southern Taiwan, and government agencies were frequently using of anti-mosquito drugs for epidemic prevention which not only costs high but also increases the evolutionary pressure of vector mosquitoes, forcing young mosquitoes or larva to develop resistance and improve their survival ability. The solution for infectious disease prevention and control is the identification of the best intervention timing to disrupt the growth cycle by applying insecticides at the right phase of disease diffusion. In such a way, the cost of control can be effective, and epidemic incidents can be controlled before sensible damages occur. This study aims to develop a forecasting method for intelligent epidemic prevention.

The growth process of arthropod-borne infectious diseases should be understood to formulate effective epidemic prevention strategies. A diffusion model of the differential equation commonly used in mathematical epidemiology is adopted in this study to predict the transmission pattern of dengue fever in the next few years based on the recorded dengue fever data in Kaohsiung from 2006 to 2016 [1, 2]. In the 10-year data from the Health Bureau of Kaohsiung Municipal Government, 896 zones of Kaohsiung are included in several years. However, few cases are reported in only 251 zones, and only district data are available in most of the years. The number of reported cases is not equivalent to the number of infected patients. In this study, this issue is addressed using a negative binomial distribution. The data obtained include not only the number of cases but also the area, population, temperature, humidity, rainfall, the population of schools, and Breteau index of adult mosquitoes in each locality. Qianzhen District, with the most dengue cases, is selected as an example. Although temperature variations in these several years are similar, the number of people infected per thousand was lower in 2014 with a high mosquito index than that in the next year with a higher mosquito index.

Regardless of the trend in diffusion time-sequence change, the year with the most dengue fever cases is used to conduct static spatial thermal zone analysis. Hausdorff distance verification shows that the area where dengue fever appears with the highest probability includes most areas in Qianzhen District, and the area with the second-highest possibility is No. 3 resident region. Spatial verification result is similar to general cognition. Therefore, the basis of this study is appropriate. On the basis of the spatial analysis result, we estimate and calibrate the diffusion model of dengue fever infection in the densest areas and the population of Kaohsiung as a whole to predict the trend of dengue fever occurred in the coming year.

The issue of intelligent epidemic prevention is extremely challenging. Assuming that only one short time of mosquito eradication can be conducted for an infinite generation of mosquitoes, we find that if mosquito eradication is carried out at the early stage of the host’s onset, the mosquitoes produced later still bite an infectious host. Since then, a mosquito vector has remained infectious for generations. However, if the timing of mosquito eradication is too late, the host population is infected. As such, epidemic prevention fails. Theoretically, if hosts are fully infected and healed, the best time point should be at the time when hosts are just healed. Therefore, the choice of this time point depends on the prevalence of hosts but not on the growth conditions of mosquitoes. The use of environmental temperature to regress the prevalence of infectious diseases is inappropriate. Infected hosts and vectors are dynamically diffusive in time and asymmetrically mobile in space. The best time point must be known through special analytical methods and lengthy and complicated operations.

Studies have been widely performed on general epidemic prevention and surveillance, as well as epidemic control, but such studies remain incomplete. Shen et al. [3] analyzed the data of a small number of clinical case reports by using the Bayes probability algorithm and proposed a time prediction model for future outbreaks of infectious diseases throughout the city. Difficulties in dengue fever epidemic prevention include the poorly described trend and dynamics of vector mosquitoes. Therefore, no complete solution has been proposed.

Andraud et al. [4] reviewed 42 dynamic models of dengue fever transmission in the time dimension and completely classified them to describe the dynamic transmission models of these vector-borne infections and their corresponding control methods. Nuraini et al. [5] discussed the transmission dynamics of dengue hemorrhagic fever caused by the cross-infection of different dengue viruses and proposed a control method to eliminate the stable balance by equilibrium solutions of two or more serotype infections.

Nishiura et al. [6] estimated the basic transmission value R_0_ and found that dengue fever is a periodic epidemic disease. This periodicity affects the transmission ability of mosquito vectors or R_0_ but does not influence environmental factors. R_0_ is also a variable related to specific environmental factors so that it can predict prevalence in the coming year. Therefore, the regression model analysis is inappropriate without considering the transmission capacity of mosquito vectors. Amaku et al. [7] and Polwiang [8] compared two kinds of vector-borne infectious models and emphasized the advantages and disadvantages of both models as the foundation of future model improvements.

Zamiri et al. [9] investigated the epidemic outbreaks in temporal and spatial patterns by an SIR nonlinear dynamic model without considering the influence of vectors. Similar to our study, they also suggest that numerical studies can help the early prediction of the epidemic in terms of peak and duration. Chang et al. [10] re-emphasized the importance of understanding the transmission dynamics and suggested the prediction of dengue epidemic can be an early warning tool. Kilicman [11] contended that dengue transmission possesses memory by analyzing the SIR epidemic model. Cahyono et al. [12] analyzed the range of parameters that lead to the stability of the dengue fever SIR model. Although the epidemic research of dengue fever applies the SIR model extensively, however, few studies can estimate parameters from the case records of real clinic reports. The insufficiency is partly due to the computational difficulty. Our study thus overcame the difficulty and obtained the parameters through an advanced Bayesian technique.

Finally, few studies have assessed dengue epidemic control. Gersovitz and Hammer [13] analyzed the economic effects of investment on epidemic prevention and assessed the cost-effectiveness of policies. Chanprasopchai et al. [14] investigated the effect of dengue vaccination through the SIR model, leaving aside the safety dispute, and suggested a significant reduction of hospitalization time. In Rio de Janeiro, 43 different insecticidal strategies were applied to adult mosquitoes and larvae for 5 years. Studies on the insecticide resistance of vector mosquitoes have shown that frequent insecticide applications are costly and may lead to the development of insecticide resistance in larvae [15].

## Results

The accuracy of the parameter estimation algorithm by using the vector-susceptible-infectious-recovered with exogeneity (VSIRX) model containing vector interaction trains with 3-year test data; the parameter estimation results satisfy our expectations.

**Fig. 1:**
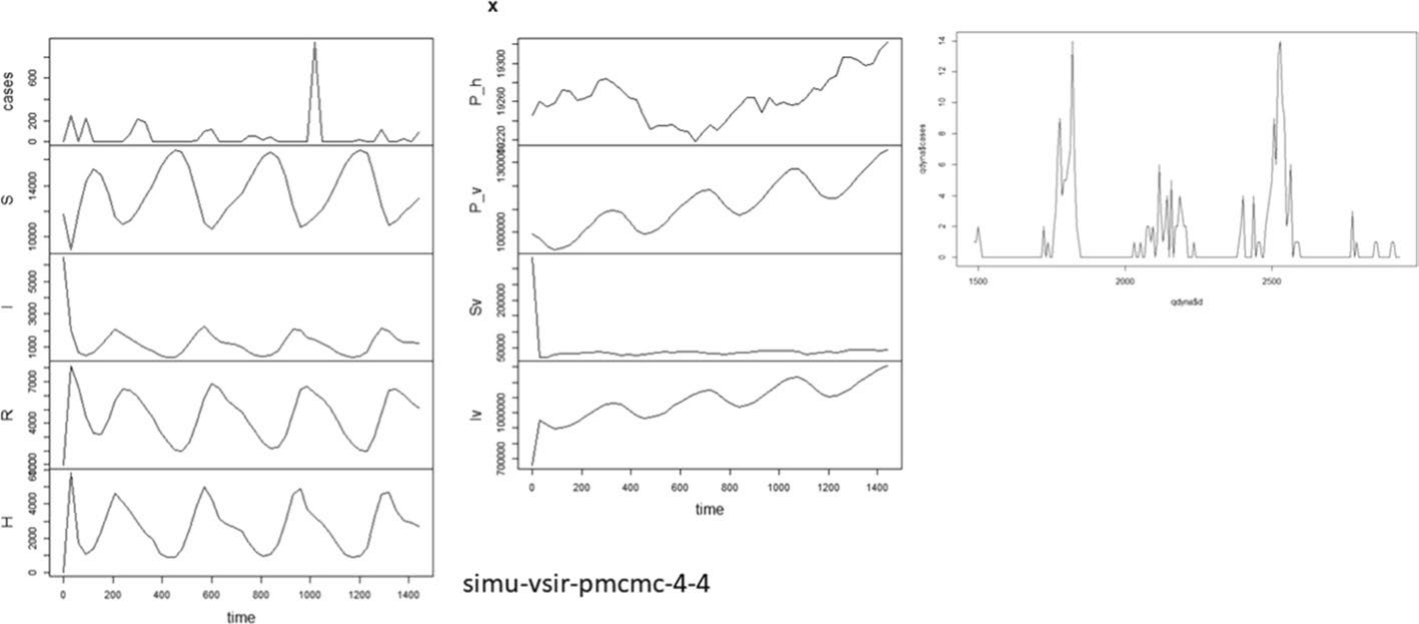
VSIRX model training with 4-year data in 2010–2013

In Fig. 1, when VSIRX model trains with 4-year data in 2010–2013, the 4-year cyclic result obtained completely meets our expectations. We then re-describe the interpretation in Fig. 1. The right figure shows the number of reported cases in 1456 days, namely, 52 weeks per year. Although factors such as temperature and vector are not included in the model, the number of infected people “H” appears periodically because of the oscillation caused by the cyclical effect. The number of reported cases predicted, the number of susceptible hosts “S”, the number of infectious hosts “I”, the number of hosts recovered “R”, and the number of accumulative infections per week “H” is shown on the left from top to bottom. The number of reported cases is a part of the cumulative number of infections per week (ρ = 0.2144). The next column includes the total population under the equivalent effect, the total vectors under equivalent effect, the number of infectious vectors, and the number of infected vectors. No overfitting phenomenon is observed in the random trajectory of the number of reported cases. A negative binomial perturbation is found in the estimated number of infected people, which is in line with the expectation of statistical estimates.

The estimated report rate is about 2%. The report rate can be correctly estimated because we can estimate a report rate with the highest possibility from the increasing trend of reports, considering that the basis for the cumulative epidemic is the actual number of infected people.

In Fig. 1, the infections of vector mosquitoes (Iv) fluctuate and increase yearly. Although the low temperature in winter reduces the growth rate of vectors, the survival of infected vectors raises the basis of dengue fever epidemic in the coming year.

**Fig. 2:**
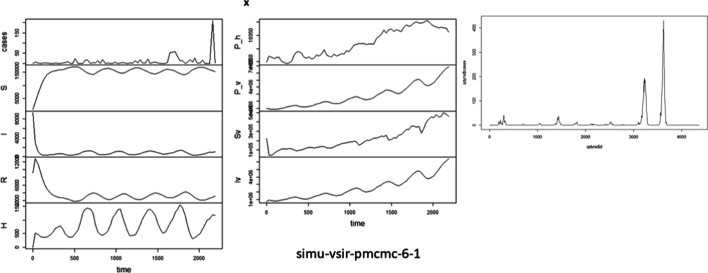
The verification results of the VSIRX model for additional testing data in 2014 and 2015

In Fig. 2, when the VSIRX model was used to verify and analyze the data in 2014 and 2015, the results obtained are consistent with the reported data. Here, to avoid the negative binomial distribution added by overfitting, we use simulation results that are not precisely the same as the data provided, and the expected statistical errors occur.

## Discussion

Followed by the previous 4-year prediction, the 6-year prediction shows the trend of verification in the last 2 years. This study shows that the proportion of vectors infected with dengue fever fluctuates because of the temperature cycle and seasonal activity. However, the survival of infected vectors accumulates the number of infected vectors in the next year so that the proportion of infected insect vectors shows an exponential growth trend every year. In Iv drawing of Fig. 2, the up-and-down increasing trend yearly can reflect the exponential growth trend. The low temperature in winter slightly affects the exponential growth of vectors, resulting in outbreaks in 2014 and 2015. From the predicted results, we can fully predict the number of reported cases and the cumulative number of unreported cases per week in the next 2 years. This parameter is critical for epidemic prevention. The possibility of increasing or decreasing according to temperature can be determined, and epidemic prevention units can be informed about the exact number of infections per day in the future and the number of hidden cases. This study suggests that the yearly cumulative effect should also be taken into account, giving exogenous causal variables. According to artificial intelligence estimation, the best time point for severe vector infection and timely medication for blocking the growth of vector mosquitoes can be determined.

Mosquito eradication can only be temporary. The temporary chance must fall at two-time points: first, many mosquitoes are infected, and second, numerous residents are infected. When an epidemic situation is not difficult, the two incidents occur one after another. After an outbreak, all people and mosquitoes are infected. As such, any compensate job will become too late. Previous epidemic prevention efforts lacked estimates of infected mosquitoes.

Nonetheless, knowing the infected residents shows a high incidence, the infected mosquitoes are infected after a sufficiently long time. Therefore, instead of choosing a time point, killing the mosquitoes seems like the only option to stop dengue fever infection. However, many years of efforts in Taiwan and other countries have shown that mosquitoes show an increased resistance, and the time difference from dosing to mosquito reproduction is becoming short. As a result of increasing evolutionary pressure, resistant mosquitoes have emerged. This study hopes to estimate when a significant population of mosquitoes is infected before a major outbreak occurs. This study also assumes that mosquitoes killed by drugs are indeed infected mosquitoes, so proliferating mosquitoes are not infected, and infected residents have passed the infection period of the virus.

Current high‐intensity insecticide strategies not only require a large amount of budget for biological control but also apply an intense evolution pressure to mosquito strains, thereby forcing their larvae to develop resistance to the insecticide for survivals. Infectious disease prevention and control aim to find the best intervention timing and places to break the growth cycle by applying insecticides at the right phase of disease diffusion. In such a way, control measures without knowing the vector growth pattern can be cost-effective, and epidemic prevalence can be controlled before sensible damages occur.

The impact of dynamic infection among vector mosquitoes is incorporated in this study, so the estimated strategy obtained by VSIRX model is accurate. Epidemic prevention must be sensible. The instinct of living things for survival should also be noted while eliminating the source of infection. If the intensity of mosquito eradication is not properly controlled, the surviving mosquito population becomes resistant and difficult to eradicate. We elucidate the status of vector-borne infections. At the best time, we can eliminate precisely and effectively mosquitoes, thereby more effectively preventing the epidemic. Accurately grasping the dynamics of vector mosquitoes by using the differential equation is similar to a special drug. In this way, the epidemic situation can quickly recede, and the goal of intelligent epidemic prevention is achieved.

## Conclusions

In this study, the problems originally proposed to be solved have been addressed, and the original goals have been achieved. A model has been established based on problem context and novel methodology development, considering energy accumulation-delayed spread-epidemic prevalence. According to different time points, the growth curve in spread gestation is continuously updated with the latest information, and the epidemic prediction model of dengue fever is established to simulate and evaluate the best chance for dengue fever prevention and control. Substantial industrial benefits can be used for policy recommendations.

This study aims to establish a basic model of the correlation between vector factors and dengue fever clustering in South Taiwan and provide a reference for establishing a time–space flow prediction model. Effect evaluation and control opportunity are simulated, and the time–space flow prediction model of dengue fever epidemic is developed to evaluate the timing of epidemic prevention and provide a reference for creating a dengue fever prevention policy.

Difficulties in preventing dengue fever epidemic include understanding the trend and dynamics of vector mosquitoes. As such, no complete solution has been established. An extensive parallel operation has also been created using high-speed computers based on mathematical dynamics (MCMC VSIRX) and a high-speed high-efficient algorithm to learn the most probable parameters in dynamic models within hours by using limited epidemic case reports, including various values, such as the incidence of disease and bite rate, which are difficult to measure directly. On the basis of the actual number of reported cases and the logic among various numbers, we can estimate the situation of vector-borne infections in each region that cannot be observed through the sampling method. The benefits of high-speed computing go beyond that.

Among the estimated parameters, an important one is the “disease report rate”. Because of the intervention of authorities, potential patients may resist receiving medical screening in order not to be isolated. The surrounding community may yield social stigma to the source person of infected. This effect of afraid-to-be-examined also appears in recently epidemic diseases. The actual report rate is key to the battle of public health, but no one can easily get his number. This study found a report rate that closed to the historical record. Therefore, the estimation can be useful information for the future work of governmental authorities.

We can also filter more than tens of billions of possible schemes and follow-up effects in just a few days based on the ability of high-speed computers and unique, efficient algorithms and determine the best time point with statistical significance for epidemic prevention units. In this manner, we can make epidemic prevention decisions.

## Methods

The statistical model for epidemiological analysis of dengue fever dissemination is based on the primitive model of Kermark and McKendrick [16], which has been widely applied for mathematical epidemiologists [4]. The population and vector mosquitoes are divided into multiple groups (compartment) with different infection status by using an SIR clustering model. In a given environment, the mathematical model of dengue fever can express the interactive growth and transmission of dengue virus between human hosts and vector mosquito hosts. The transfer parameters of the environment determine that the model can flexibly state the effect of the interactive infection and growth of human–vector mosquito population.

**Fig. 3:**
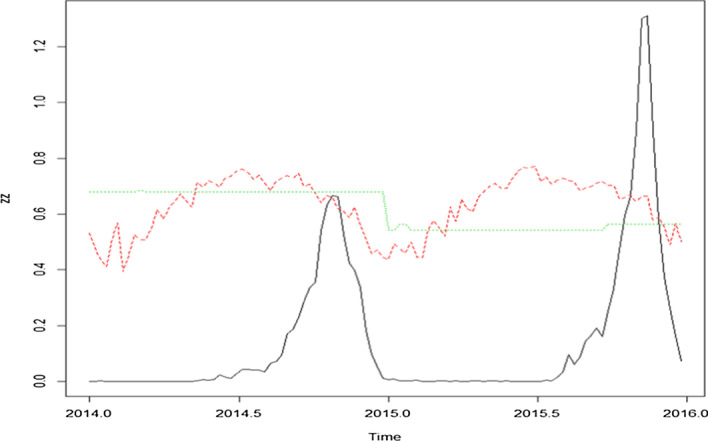
The dengue cases per 10 thousand capita, temperature, mosquito index, in black, red, and green lines, respectively

To ease the explanation of our VSIRX model [Eq. (1)] [17], a real figure was drawn from 2014 and 2015, as shown in Fig. 3, where the solid line represents the number of cases; the dash line represents the temperature, and the dotted line represents Breteau index. In Fig. 3, we can observe that the temperature variations in the two years were similar and the mosquito index at the first year was higher than that of the second year, but, inversely, the number of people infected per 10 thousand was lower in 2014 than the next year. Our differential equation model in Eq. (1) can easily explain this accumulation phenomenon. The variables capturing the population size in each infection status are denoted by the letters *S*_*H*_*, I*_*H*_, and *R*_*H*_, where “*S*_*H*_” is the number of people susceptible, i.e., who can be infected, “*I*_*H*_” is the number of people infected, and “*R*_*H*_” is the number of people recovered. The model also includes the vector activities, and (*S*_*v*_, *I*_*v*_) is the number of Infectious and infected vectors, respectively. The number of recovered vectors is not included because the life cycle of mosquitoes is not long enough to be modelled. The total number of people (*S*_*H*_ + *I*_*H*_ + *R*_*H*_) likely remains unchanged in the system because of the low mortality rate caused by dengue fever. Each individual has also assumed has an equal chance of being bitten by vector mosquitoes in a closed system. The definition and initial values of parameters in Eq. (1) can be found in Table 1.

1 $$\left\{ {\begin{array}{*{20}l} {\frac{{dS_{H} }}{dt} = - rb_{H} I_{M} S_{H} - \mu_{H} S_{H} + \sigma_{H} R_{H} + \Lambda_{H} ,} \hfill \\ {\frac{{dI_{H} }}{dt} = rb_{H} I_{M} S_{H} - \left( {\mu_{H} + \gamma_{H} } \right)I_{H} ,} \hfill \\ {\frac{{dR_{H} }}{dt} = \gamma_{H} I_{H} - \left( {\mu_{H} + \sigma_{H} } \right)R_{H} ,} \hfill \\ {\frac{{dS_{v} }}{dt} = - rc_{v} I_{H} S_{v} - \mu_{v} S_{v} + \Lambda_{v} ,} \hfill \\ {\frac{{dI_{v} }}{dt} = rc_{v} I_{H} S_{v} - \mu_{v} I_{v} .} \hfill \\ \end{array} } \right.$$

**Table 1:** Initial values of parameter estimated

Parameters	Description	Initial value	Assumption
	Unit of simulation time scale	1	Day
	Unit of parameter timebase		Year
${{a}}$	Rate host on vector bite	0.5	Bites per day
${{{b}}}_{{{H}}}$	Rate host on infectious bite	0.5	Positive per bite
${{{\mu}}}_{{{H}}}$	Rate host nature mortality	5.5 × 10^–5^	Dead ratio per day/50-year life
${{{\gamma}}}_{{{H}}}$	Rate host recovery	1 × 10^–2^	Recovered ratio per day/3 month
${{{\eta}}}_{{{H}}}$	Rate host to vector infection	0.375	Probability of infection
${{{\sigma}}}_{{{H}}}$	Rate host immunity loss	1 × 10^–3^	Loss ratio per day
${{{\Lambda}}}_{{{H}}}$	Rate host growth	10	Numbers per day
${{{c}}}_{{{M}}}$	Rate vector on infectious host bite	0.8	Bites per day
${{{\mu}}}_{{{M}}}$	Rate vector nature mortality	0.143	Dead ratio per day/1-week life span
${{{\eta}}}_{{{M}}}$	Rate vector to host infection	0.75	Probability of infection
${{{\Lambda}}}_{{{M}}}$	Rate vector growth	1000	Numbers increase per day/hatch rate should be time-variant
${{r}}$	$a/{N}_{H}$ rate bite per mosquito per human		
${{{N}}}_{{{H}}}$	Host population size	1020	

The numbers between the infected and official recorded may not be consistent. Our model must capture the inconsistency. The counting process *C(t*_*1*_*, t*_*2*_*)* is the number of officially reported cases between times *t*_*1*_ and *t*_*2*_. The number of people actually infected between times *t*_*1*_ and *t*_*2*_ is *ΔN*_SI_*(t1, t2*). We, therefore, associate the two numbers with relations of proportion and uncertainty in [Eq. (2)].

2 $$C\left( {t_{1} , \, t_{2} } \right) \, \sim \, NegBin \, (\rho \Delta N_{SI} \left( {t1, \, t2} \right),\theta )$$

The proportion of infected to be reported to authorities is denoted as the report rate *ρ.* The distribution *NegBin* (number = n, prob = *θ*) is a discrete negative binomial probability distribution of the number of successes (with probability *θ*) in a sequence of independent and identically distributed Bernoulli trials before a specified number of failures (n) occurs.

Obtaining data about viruses in insects is limited by various factors. However, we still have the track of human disease. We can still reversely infer the most-possible infection trajectory of insect vectors from the result. On the basis of the actual number of cases reported and the logic among various numbers, we estimate the situation of vector-borne infections in each region that cannot be observed by sampling observing method. On the basis of dengue fever case data, we can estimate a set of the most-possible parameters in a half-day of computation using the most advanced parameter estimation algorithm and a 32-core high-speed computer. We can easily verify the results of the analysis with the worst outbreak starting from Kaohsiung in 2014 and 2015. Our primary analysis result shows that the estimated parameters are sufficient to represent the current pattern of dengue epidemic spread [18]. After repeated calibration by performing Markov chain Montel Carlo (MCMC), and the estimation are carried out, the set of parameters is obtained in accordance with the trajectory of the reported cases.

## Data Availability

The dengue fever case data were provided from Taiwan Center for Disease Control. The dataset can be downloaded in csv or xls formats. https://nidss.cdc.gov.tw/en/nndss/disease?id=061

## Abbreviations

SIR: Susceptible-infectious-recovered
VSIRX: Vector-susceptible-infectious-recovered with exogeneity
MCMC: Markov chain Montel Carlo
BEM: Boundary element method
